# Adoption Does Not Increase the Risk of Mortality among Taiwanese Girls in a Longitudinal Analysis

**DOI:** 10.1371/journal.pone.0122867

**Published:** 2015-04-29

**Authors:** Siobhán M. Mattison, Melissa J. Brown, Bruce Floyd, Marcus W. Feldman

**Affiliations:** 1 Department of Biology and Women’s, Gender, and Sexuality Studies Program, Boston University, 5 Cummington Mall, Boston, Massachusetts, 02215, United States of America; 2 Department of Anthropology, University of Auckland, Private Bag 92019, Auckland Centre, 1142, New Zealand; 3 Harvard-Yenching Institute, 2 Divinity Ave, Cambridge, Massachusetts, 02138, United States of America; 4 Minnesota Population Center, University of Minnesota-Twin Cities, 50 Willey Hall, 225 – 19^th^ Avenue South, Minneapolis, Minnesota, 55455, United States of America; 5 Department of Biology and Morrison Institute for Population and Resource Studies, Stanford University, Stanford, California, 94305, United States of America; Durham University, UNITED KINGDOM

## Abstract

Adopted children often experience health and well-being disadvantages compared to biological children remaining in their natal households. The degree of genetic relatedness is thought to mediate the level of parental investment in children, leading to poorer outcomes of biologically unrelated children. We explore whether mortality is related to adoption in a historical Taiwanese population where adoption rarely occurred among kin. Using Cox proportional hazards models in which adoption is included as a time-dependent covariate, we show that adoption of girls does not increase the risk of mortality, as previously suggested; in fact, it is either *protective* or neutral with respect to mortality. These results suggest that socio-structural variables may produce positive outcomes for adopted children, even compared to biological children who remain in the care of their parents.

## Introduction

Adoption and fostering have often been viewed negatively against the “ideal type” of nuclear family most common in Western populations (e.g., [1]), in part because adoptive children have often been shown to suffer disadvantages in health and well-being compared to biological children (e.g., [2,3]). Long considered paradoxical in evolutionary social science (i.e., because it involves expending effort on genetically unrelated individuals) [4], more recent theoretical and empirical treatments of delegated parenting have pointed to the benefits that may accrue as a result of adoption (e.g., [5–8]), including inclusive fitness benefits resulting from rearing adoptive children who are genetically related to their adoptive parents [6,9]. Adoption has been hypothesized to benefit adoptive parents, adopted children, and biological parents, depending on the specific circumstances surrounding adoption. Hypothesized benefits include lineage continuity [10–13], labor and old-age support [7,11,14,15], improved familial or social alliances [11,14,15], and enhanced social mobility of adoptive children who lack relevant opportunities in their natal households [6,8]. Where such benefits are not apparent, adopting a child represents a net cost to adoptive parents, and adoptive children are not expected to fare as well as their biological counterparts. In this paper, we explore the relationship between adoption and mortality in a society where adoption was not usually conducted among close genetic relatives and where ethnographic evidence (e.g., [14]) suggests that adoptive children were maltreated. Based on this evidence we initially hypothesized that adoptive children would suffer higher mortality than biological children raised in their natal households, but we found the opposite. We now introduce our study site and discuss more generally how socio-structural contexts give rise to alternative explanations for adoptive behavior and its outcomes.

Our study examines the consequences of adoption on mortality in Taiwan during the period of Japanese colonial administration (1895–1945). Adoptions were frequent during this period, occurring in households across the socio-economic spectrum [14,16], and served various purposes within the normative system. According to Wolf and Huang [14], male children from agnate lineages (*kepangkia;* 过房子) could be adopted as heirs when biological sons were lacking. In such cases, they inherited all the rights and duties of biological sons and maintained linkages to their birth parents. In contrast, a son adopted from strangers as a *bieng-lieng-kia* (螟蛉子) was more expensive and considered the property of the adoptive household. Girls were adopted either as adopted daughters (AD/*iong-lu/*养女) or adopted daughters-in-law (ADIL/*simpua/*媳妇仔), based on family circumstances. Adoptions as daughters occurred in cases where no sons had been born, in the hopeful belief that adoption of a girl would “lead in” the birth of a son (“therapeutic” adoption) and with the practical understanding that an AD could, if necessary, stand in for a son. Adoption of a daughter-in-law was necessary for the “minor” form of marriage, which involved rearing a genetically unrelated girl as a future bride for a co-resident boy who was one of the sons of the household. Finally, slave girls (*cabokan/*婢女*)* were less commonly purchased in relatively wealthy households (the practice was outlawed by 1916).

Minor marriage constituted the majority of adoption cases during the Japanese colonial administration of Taiwan [15,17] and these adoptions typically occurred in the very early months or years of an ADIL’s life [14,16]. Ethnographers such as Wolf [14,15] have linked minor marriage with several motivating factors, including maintaining harmony within the family (i.e., because minor marriage avoided the strains that could emerge between mothers-in-law and adult daughters-in-law), economic considerations (i.e., avoiding the costs associated with adult marriages), and the exchange of an unwanted biological daughter with an eventual wife for a biological son. By contrast, the “major” form of marriage involved the transferal of a physically mature young woman with ceremonial feasting and exchange of money and goods to her husband’s home. The costs of major marriage were both financial and social. They included a significant brideprice on the part of the groom’s family, possibly a dowry on the part of the bride’s family, an extravagant marriage ceremony, and tensions between mother-in-law and daughter-in-law. Social strains were said to be avoided by minor marriages because adopted daughters-in-law were reared and often breastfed by their mothers-in-law and ADIL were often adopted from villages outside the adoptive household’s community, thus avoiding protective intervention by the adoptive child’s natal family [16]. Their ubiquity notwithstanding, minor marriages were considered by Taiwanese to be socially inferior to major marriages. Scholars have also associated minor marriage with several disadvantages, most notably a failure to create strong conjugal alliances and reduced fertility when husbands and wives were co-reared from very young ages [14,15]. Ethnographic evidence also suggests that ADIL were routinely beaten and otherwise maltreated by their adoptive families [14,15].

Poor treatment of adopted children relative to biological children is consistent with evolutionary theory that invokes kin selection. Briefly, inclusive fitness (i.e., kin selection) theory predicts that if the costs (*c*) and benefits (*b*) of investment are held constant, parents will bias investment according to their degree of genetic relatedness (*r*) to the children in which they invest [18]. Kin selection has been invoked to explain why parents often prefer to foster or adopt more closely related children [19–21], yet measures of parental investment (PI) and its associated outcomes do not always conform to predictions from studies employing this perspective (e.g., see [22]). Several studies support the role of genetic relatedness in structuring PI [23–25]. In a series of famous studies, step-children were shown more likely to suffer abuse, neglect, and murder than biological children [3,15,26–28]. The degree of relatedness was positively associated with child perceptions of received care within families [25] and in emotional proximity [29] in two USA-based samples. All else being equal, Xhosa fathers [30] and American fathers [31] also invested more intensively in their genetic offspring. In contrast, in one USA sample, children fostered among kin fared similarly to children fostered to non-kin [22] and a comprehensive review of fosterage studies has shown many detrimental outcomes of children fostered by kin [32]. Finally, social fathers engaged as often or more often than biological fathers with their 5-year-old children surveyed in the Fragile Families and Wellbeing Study [23].

These inconsistent results underscore that kin selection theory is but one mechanism that might influence the evolution of adoption [6,8,33]. Moreover, different constraints and motivations affecting adoption are associated with differences in PI and outcomes. For example, where infertile parents adopt un-related children, adopted children may fare as well as biological ones. Indeed, Case et al. [34] have shown that fostered, adopted, and step-children obtained less education than biological children, but only when they were co-reared with biological siblings. In contrast, Gibson [24] has shown that adopted children received similar or more PI compared to genetic children residing in the same households among United States parents. He argued that deliberate adoption to avoid stigma associated with small family size may have encouraged high levels of PI in these parents. Similarly, Berger et al. [23] and Anderson et al. [30,35] have shown high levels of step-father support of non-biological children, seemingly motivated by relationship effort (i.e., the effort expended toward obtaining or keeping a mate), rather than parental effort, per se, while Borders et al. [36] found no differences between outcomes of adoptive and biological families in a matched sample of USA families. Silk has argued extensively that economic considerations are crucial motives of adoption in many settings [6,7,19–21]. Taken together, these studies reinforce the conclusion that the cultural and ecological (e.g., social and structural) contexts surrounding adoption are critical determinants of its frequency, underlying motivations, and health and affective outcomes [1,2].

Though we know little about adoption outcomes in non-Western settings [2], accounts of historical Taiwanese and broader Chinese adoption systems have largely corroborated the kin selection hypothesis. As mentioned above, whether adoptions were undertaken to perpetuate the lineage or for other reasons, Chinese and Taiwanese viewed them negatively relative to more “natural” means of raising children [12]. Girls were easily given up for adoption in China [12,14,37] where strong son preference was the norm [14,15,37], in association with the view that daughters were others’ ancestors. Indeed, the probability of giving away a daughter for adoption was as high as 70% by age 15 in northern parts of Taiwan in the early 20^th^ century [14] (p.233). Arthur Wolf and others [14,15,38–40] have hypothesized that minor marriage, while frequently chosen as a form of marriage because it avoided the costs and within-household tensions inherent to major marriage, was detrimental to adopted daughters, who suffered nutritional deprivation, beatings, and higher mortality. Though previous accounts of this system hypothesized and indeed found worse outcomes, including higher mortality, for ADIL relative to their biological counterparts [14,15,41], they were based on data that were somewhat limited geographically and ethnically, and/or did not control for important covariates known to mediate the relationship between mortality and adoption in this population. We test the kin-selection hypothesis here, performing a longitudinal analysis of a larger set of data from 13 sites distributed over much of Taiwan and a total sample size of up to 74,692 individuals (Table 1D and S1 Fig—map of study sites), controlling for important covariates (e.g., locale, sex of child, parity, socioeconomic status), and including adoption as a time-dependent covariate in order to attenuate the potential selection bias that results from the possibility of individuals dying before being adopted [9,14,15].

**Table 1:** Descriptive statistics by gender^a^.

	Number of Mean in Total Population (% of total or SD)	Male	Female
Death	20,152 (27.0%)	11,183 (28.9%)	8,969 (24.9%)
Age at Death (years)	3.01 (6.21)	3.48 (7.06)	2.43 (4.89)
Sex	74,692 (100%)	38,679 (51.8%)	36,013 (48.2%)
Adopted	8,469 (11.3%)	1,343 (3.47%)	7,126 (19.8%)
Household Head Occupation^b^			
Agriculture	37,755 (59.2%)	19,501 (59.0%)	18,254 (59.3%)
Laborer	13,237 (20.7%)	6,857 (20.8%)	6,380 (20.7%)
Craftsman	1,869 (2.9%)	977 (3.0%)	892 (2.9%)
Merchant	10,471 (16.4%)	5,457 (16.5%)	5,014 (16.3%)
Landlord	471 (0.7%)	239 (0.7%)	232 (0.8%)
Birth Cohort			
1 (1906–1915)	13,742 (18.4%)	7,252 (18.7%)	6,490 (18.0%)
2 (1916–1925)	15,485 (20.7%)	7,889 (20.4%)	7,596 (21.1%)
3 (1926–1935)	21,316 (28.5%)	12,040 (28.5%)	10,276 (28.5%)
4 (1936–1945)	24,149 (32.3%)	12,498 (32.3%)	11,651 (32.4%)
Number of Live Siblings in Natal Household^b^	2.89 (1.91)	2.89 (1.92)	2.90 (1.91)
Uxorilocal Residence at Birth	8,700 (11.6%)	4,488 (11.6%)	4,212 (11.7%)
Illegitimate	4,756 (6.4%)	2,431 (6.3%)	2,325 (6.5%)
Age at Adoption (years)	2.40 (3.30)	2.92 (4.24)	2.30 (3.08)

^a^Denominators in percentages differ slightly among variables due to missing data.

^b^See definitions in Methods, above.

## Materials and Methods

### Data

Our data come from a computerized sample of Taiwan’s household registers spanning 40 years (1906–45) during the Japanese colonial period. Taiwan’s population at that time was predominantly ethnic Chinese and rural [41–43]. The colonial government used the household registration system to monitor Taiwanese society: households were supposed to report all relevant demographic changes (births, adoptions, marriages, deaths, migration, etc.) to their local police station within 10 days of their occurrence, and local police visited every household at least twice each year to verify registration; punishments for failure to report were severe [15,41,44]. The colonial-period registers are renowned for their accuracy [e.g., 42,45], having been checked using direct and indirect demographic methods [41,46] and through retrospective interviews of individuals enumerated in the registers, particularly for under-reporting of girls’ deaths [43]. Note that differentiation of girls adopted as daughters versus daughters-in-law was not explicitly reported in the registers, but must be inferred from family structure (e.g., the presence of a son immediately prior to the adoption of a girl strongly implies adoption for the purpose of minor marriage) [14,15,43,44]. The total number of unique individuals in the data set analyzed here was 74,692 and the total number of death events among these was 20,152. Age-specific mortality rates and life tables were constructed using this sample. Event history analyses made use of multiple records of the same individuals over time so that the sample of records was larger. The number of individuals and death events represented in a given analysis differed according to completeness of information as individuals with missing values in any variable were removed from a given analysis.

### Descriptive Analyses

All analyses were conducted in R, version 2.15.2 [47]. Our descriptive analyses included calculation of age-specific mortality rates (ASMRs) as the number of death events occurring during a given age interval from time *t*_*0*_ to time *t*_*1*_, divided by the person-years of exposure to risk of death for that age interval. ASMRs thus measure aggregate mortality in a given age interval and can be used to explore data for changes in mortality from one age to the next. The observed ASMRs formed the basis of life tables generated using standard methods [48]. The life tables model the aggregate effects of age-specific mortality patterns on related outcomes, and are of interest here primarily in terms of how they affect the life expectancy at each age. Because only individuals who were born during the period of observation (1906–45) and born within our study sites were considered, ASMRs generated via our own data were truncated after age 40. We thus supplemented our data with mortality rates from UN model life tables (Far Eastern patterns chosen to match observed ASMRs as closely as possible). In other words, all values of _*n*_ *m*_*x*_ (the mortality rate from age *x* to age *x* + *n*) above age 40 in our life tables were taken from UN model life tables.

### Inferential Analyses

To explore the association between adoption status and mortality, we employed Cox proportional hazards (CPH) models, which include covariates, potential confounding variables, and censoring due to unobserved deaths (i.e., deaths that occurred outside the observation window). Adoption status, the primary independent variable of interest, was modeled as a time-dependent covariate. Whereas longitudinal analyses of adoption outcomes allow for inspection of causality relative to cross-sectional data [2], they are not sufficient to overcome selection biases resulting from early death of would-be adoptees [9,14,17]. Survival analysis with time-dependent covariates overcomes the selection problem inherent to fixed covariates models by pinpointing the precise interval during which individuals were adopted and assessing the pooled effects of adoption and mortality within each time period [49]. The influence of adoption status as a time-dependent covariate on the hazard of mortality was thus restricted to the time-periods over which it occurred. Because our analyses break the dataset into 40 year-long intervals, the possibility of a selection bias in the first year of life remains even in time-dependent covariates analysis. We thus restricted the main analyses to individuals who had survived to at least age 0.5 years, by which time mortality had begun to drop off. In the analyses reported here, both age and adoption are modeled as time-dependent covariates, with all other variables held at fixed values. We present results from the model including all individuals from birth in (S4 Table) for comparison. We also include a model exploring mortality through age 15, the age at which major marriages typically became feasible, in order to exclude the possibility that a woman’s departure from her natal household explained the results observed at all ages (S5 Table). Additionally, the analysis presented in the main text does not model the effects of adoption status and covariates separately for males and females; we present results of these separate analyses in S6 Table.

### Variables

Even though patriliny and virilocal post-marital residence were dominant in colonial-era Taiwan, daughters could take the place of sons via uxorilocal marriages, providing both labor and descendants for households [14,15,17,50,51]. Given that uxorilocal marriage has been associated with low socioeconomic status and high child mortality in Taiwan and China [17,50,51], we include “uxorilocal” as a predictor in our models, where 1 indicates that an individual’s parents were married uxorilocally at the time of an individual’s birth; all other post-marital arrangements were virilocal and were coded as 0. Whether an individual was considered illegitimate at the time of birth was recorded in the registers and is included here as a binary variable where 1 indicates illegitimacy and 0 otherwise.

The household head’s occupation was also recorded at the time he (or more rarely, she) succeeded to the headship of the household (it was not subsequently updated), and we use it here as a general marker of socioeconomic status (see also [52]). It is treated as a fixed covariate in our models (see above), where the head’s occupation was taken at the time nearest to the birth of the indexed individual. In other words, this variable is meant to account for the contribution to mortality of the effect of differences in early-life socioeconomic status; future analyses are planned in order to assess how *changes* in socio-demographic characteristics affected the outcomes of adoption in this society. We collapsed the original (over 200) categories coded in the registers into 5 [14]: agriculture, indicating any form of employment in the agricultural sector (including owner-operator farmers, tenant farmers, and petty landlords, who worked some of their land and rented out another portion of their land); laborer; craftsman; merchant; and landlord. These broad categories were used to provide a crude proxy of socio-economic status: although the registers distinguished between a barber, tailor, and tofu-seller, for example, they did not consistently distinguish farmers who owned from those who rented their land; thus aggregate categories were deemed more useful.

The number of living full siblings (Table 1) who were born from 1906–1945 and within the study sites included in the data was also treated as a fixed covariate. This method of counting siblings may miss those who were born elsewhere (and died or left the household prior to being enumerated in the dataset). The data contained in the registers included only information on same-sex sibling order at birth, which we deemed less useful and comparable than the order of siblings alive and living in the individual’s household at the time of birth, following evidence in [14,15]. Living birth order (Table 2 and S4–S6 Tables) adds 1 to the number of living older siblings (Table 1). The cohort in which an individual was born is included as a categorical variable where 1 = 1906–1915; 2 = 1916–1925; 3 = 1926–1935; and 4 = 1936–1945. We include all cohorts in all relevant analyses, despite the inability of later cohorts to contribute to mortality at relatively advanced ages. Because the majority of deaths occurred during the first years of life, we deemed it useful to include these cohorts to enhance precision of those mortality estimates. Adoption as daughters (AD) or daughters-in-law (ADIL) was not specified in the registers, so we used an indirect indicator of the proportion of ADIL in our sample. Minor marriages were much more common in northern Taiwan than in other locales [17,44]; we thus include the level of adoption for the purposes of minor marriage (a potential precision variable in our models) as a categorical variable where 1 indicates lowest prevalence, 2 indicates moderate prevalence, and 3 indicates high (including Penghu, highest) prevalence of minor marriage.

**Table 2:** The effects of covariates on the instantaneous hazard of mortality§,*.

	Beta^a^	SE^b^	p
Adopted	-0.13	0.13	0.367
Gender^c^	0.47	0.04	0.195
Age	-0.24	0.00	<0.001***
Age^-2^	8.71	0.13	<0.001***
Living birth order	0.01	0.00	<0.001***
Craftsman^d^	-0.05	0.06	0.386
Laborer^d^	0.06	0.03	0.018*
Landlord^d^	-0.10	0.10	0.338
Merchant^d^	-0.06	0.02	0.028*
Uxorilocal^e^	0.04	0.03	0.203
Illegitimate^f^	0.06	0.05	0.156
Moderate minor marriage^g^	-0.05	0.03	0.037*
High minor marriage^g^	-0.28	0.02	<0.001***
Birth cohort = 2^h^	-0.04	0.05	0.432
Birth cohort = 3^h^	-0.05	0.08	0.526
Birth cohort = 4^h^	0.10	0.12	0.416
Adopted x cohort 2	-0.13	0.11	0.221
Adopted x cohort 3	-0.39	0.13	0.002**
Adopted x cohort 4	-0.14	0.21	0.507
Gender(M) x living birth order	-0.00^i^	0.00	0.062
Gender(M) x adopted	0.25	0.15	0.031*
Adopted x moderate minor marriage	-0.14	0.14	0.331
Adopted x high minor marriage	-0.18	0.13	0.147

^§^Number of death events = 10,963; number of records = 648,237; sample consists of individuals born under observation and surviving to at least 6 months of age.

*p-value ≤0.05,

**≤0.01,

***≤0.001. n is reduced compared to total sample due to missingness.

^a^Beta is the estimated coefficient of the relationship between a given independent variable (e.g., gender) and the outcome of interest (here, the log hazard of mortality); i.e., a one-unit change in the independent variable is associated with a Beta increase in the log hazard of dying at any time.

^b^SE is standard error of the estimated Beta.

^c^Reference category is female.

^d^Reference category for head of household’s occupation is agriculture.

^e^Reference category is not-uxorilocally married.

^f^Reference category is legitimate.

^g^Reference category is low prevalence of minor marriage; based on S1 Table; see Supplementary Methods for details.

^h^Reference category is birth cohort 1; see S1 Table.

^i^Coefficient = -8.66 x 10^–3^.

Our main interest is in how an individual’s gender and adoption status affected the likelihood of mortality. Mortality in Taiwan at this time period was high [46,53,54], as reflected in the number of observed death events (20,152) in our dataset. Adoption status is indicated in the registers, including whether the individual was adopted “out” to another household or adopted “in” by a household. Here, we consider only those individuals who were born in households in our sample and adopted out—this allows us to follow an individual from birth and removes an important selection bias that might occur if we included individuals who were adopted into the study sites from households not included in our sample (see below). Moreover, because we only follow individuals who were born “under observation” in the dataset, there is no possibility of misidentifying an adopted individual (i.e., who was adopted in) as a biological child. Both males and females were adopted, but adopted sons and daughters may have been treated differently [11,12,14,15,44]. We therefore include interaction terms for gender-by-adoption status to allow for differing effects of adoption on mortality by gender. We also include interaction terms for adoption-by-cohort, to explore whether birth cohort mediated the effect of adoption on mortality risk, as minor marriage frequency declined over time [14,15,17] and public health improved [54]. An interaction term for gender-by-living-birth-order is included to allow for the fact that son preference may be increasingly likely to be displayed at higher birth orders [14,15,55]. Finally, though we have no direct indications of whether girls were adopted as daughters or as daughters-in-law, we attempted to glean whether specific forms of adoption underlie our results via inclusion of an interaction term for adoption status-by-prevalence. If minor marriage had been predominantly to blame for high mortality rates of adopted girls [15], then mortality should have been higher for adopted girls residing in areas where minor marriage was most prevalent.

## Results

### Descriptive Analyses

**Fig 1:**
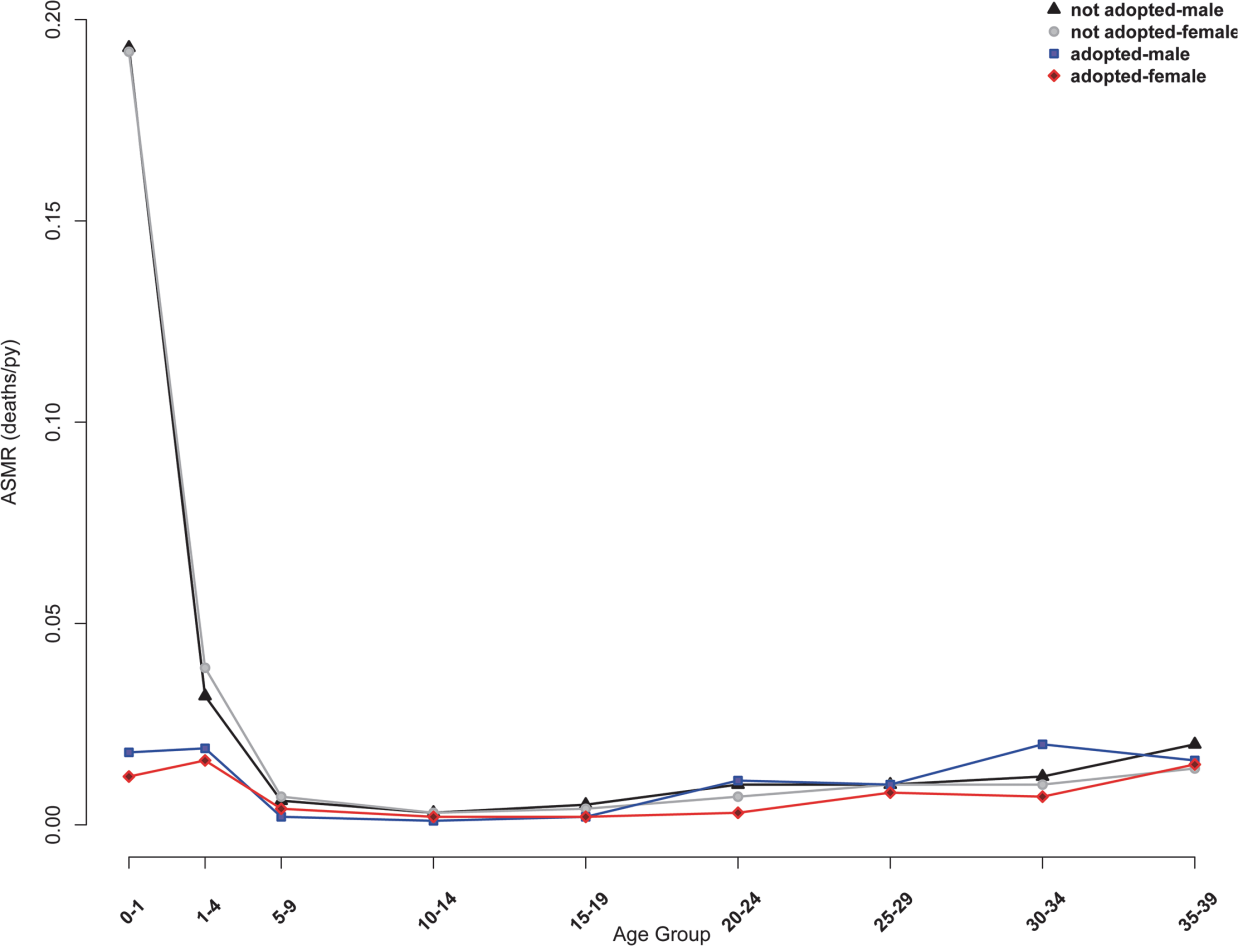
Age-specific mortality rates (ASMRs), by gender and adoption status. Unadjusted mortality shows an early life mortality advantage in both adopted males and females compared to biological children reared in their natal households. The advantage persists into adulthood for adopted females, but adopted males show slightly elevated mortality patterns in early and middle-adulthood.

**Fig 2:**
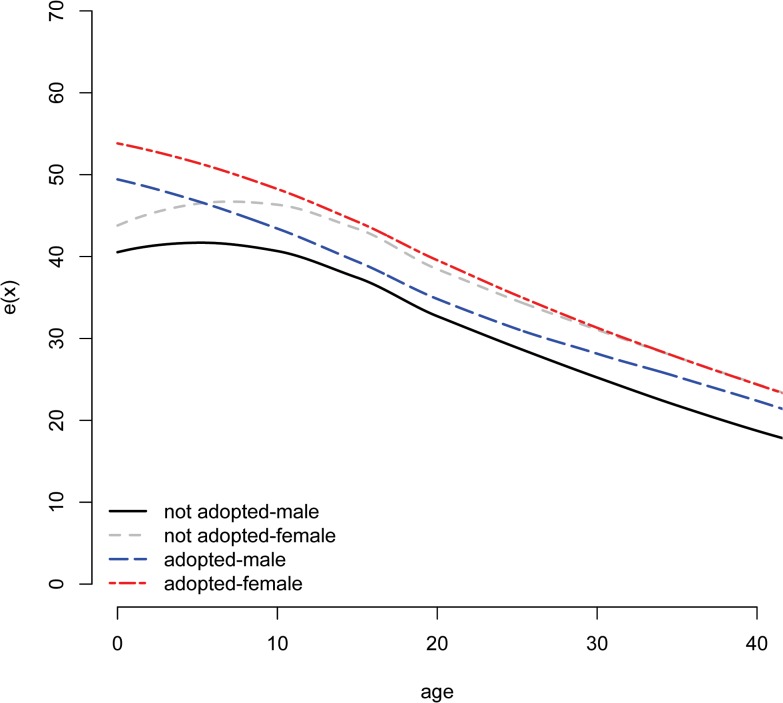
Life expectancy, by gender and adoption status. The divergence in life expectancies between females who were adopted and other categories of individuals is most apparent early in the life course. Higher life expectancies at birth for both adopted males and females suggest the existence of a selection effect, but cannot explain the persistent advantage evident among adopted children, especially girls. The curves are smoothed via loess.

Consistent with previous reports [41,46,53], we found that mortality was high and adoption frequent in Taiwan over the observed time period (Table 1). Mortality and adoption were not distributed evenly, however. Roughly five times more daughters than sons were adopted out (Table 1) and non-adopted children were approximately 4.5 times more likely to die (N = 19,589; 29.6% of non-biological children) than children who were adopted out (N = 563; 6.65% of adopted children). Predictably, mortality was highest at youngest ages, especially under 1 year of age (Fig 1 and S2 Table) and, consistent with previous reports [14,15,41], a majority of adoptions (59.4%) occurred during this high-risk period. The effects of these differences at the population level were lower life expectancies by approximately 6 years at age 1 for females who were not adopted (*e*_*1*_ = 46.8) compared to females who were (*e*_*1*_ = 53.1; S3 Table and Fig 2). (We use *e*_*1*_, rather than *e*_*0*_, to illustrate the magnitude of these descriptive differences in life expectancy as the life tables do not control for the selection effect, which is most likely to exert its influence under age 1.) This advantage persisted into and beyond adolescence for girls (Fig 1), suggesting that a selection bias exists, evident in high life expectancies at very young ages (Fig 2), but does not fully explain the relationship observed between adoption and mortality.

### Inferential Analyses

**Fig 3:**
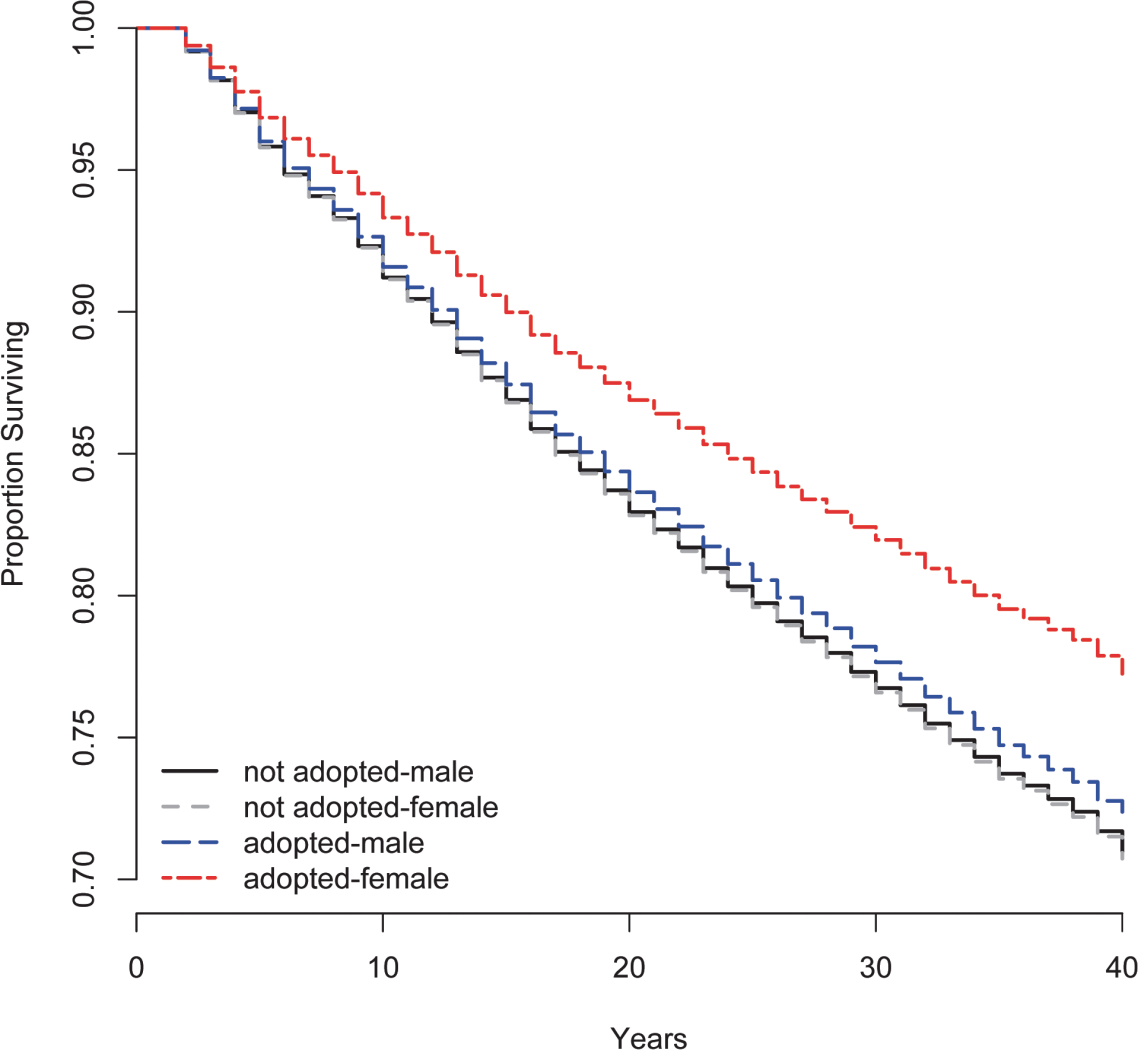
Survival plots, by gender and adoption status. Adoption status is significantly associated with predicted survivorship, but only for adopted girls. Confidence intervals have been eliminated from the figure as they obscure the main results, but do not overlap for adopted girls, only.

Cox proportional hazards models estimating the effects of age, gender, and other covariates on the hazard of mortality in our dataset confirmed relationships apparent in the above descriptions. These analyses revealed that adoption was significantly and inversely associated with mortality, but only for girls (Table 2 and S4 Table-all ages). This effect was dramatic: Fig 3 shows that roughly 78% of adopted girls could be expected to survive 40 years, compared to approximately 72% of non-adopted boys and girls. Furthermore, adoption became more protective over the duration of the study (i.e., with calendar time), even after its prevalence lessened. Our results provide partial support for the suggestion that it was ADIL, not AD, whose survivorship was highest, since the effect of adoption on mortality was strongest in areas where ADIL (minor marriage) was highest: although the interaction term was not significant (Table 2, cf. S4 Table), regions in which minor marriage prevalence was high experienced lower mortality than those where the prevalence of minor marriage was lower [17,53]. However, the effect was present for both males and females when models were run separately for each gender (S6 Table); the specific means by which male adoptees may have benefitted from a high prevalence of minor marriage is unclear. The north-south gradient of increasing mortality in Taiwan [41] does not fully overlap with prevalence of ADIL and cannot account for the separate effect of adoption on mortality. Other results of these analyses are in the anticipated directions [41]: age was the strongest contributor to the hazard of mortality; high socio-economic status (as indicated by the head of household’s occupation) relative to employment in agriculture generally was associated with lower mortality; and higher parity increased the hazard of mortality.

Because our analyses were motivated by the effects of parental investment (PI) on the outcomes of adopted versus biological children, we replicated our model on a population in which individuals under the age of 0.5 years and over the age of 15 years were excluded. This was an attempt to eliminate the possibility associated with major marriage that the effects of affines (i.e., after a biological daughter had moved from her natal home to join her husband) were included in the estimation of mortality risk. Results from this model differed slightly from the above (S5 Table). Most notably, the effects of gender and adoption on the risk of mortality were not significant; adoption maintained a protective effect in association with calendar time (i.e., it became more protective over time) and the prevalence of minor marriage was associated with a stronger protective effect. This could indicate that the protective effect of adoption is caused by mortality at later ages. The lack of statistical significance could also be due to a smaller sample size when individuals at more advanced ages are excluded. In either case, adoption is at least neutral with respect to the risk of mortality.

We also modeled the effects of adoption and covariates on the hazard of mortality for males and females separately to allow for the possibility that covariates and interactions differed by gender in magnitude and significance. These results (S6 Table) show roughly similar effects across genders, with two notable exceptions: living birth order and uxorilocal parental residence increased the risk of mortality for girls, but not for boys. In addition, the effects of socioeconomic status, using head of household’s occupation as a proxy, were inconsistent across genders—residing with a head of household who was a laborer increased the risk of mortality for boys, whereas residing with a merchant decreased the hazard of mortality for girls. In separate models, neither girls nor boys experienced protective effects of adoption with increasing prevalence of minor marriage as in the aggregate model (Table 2), and adoption was neutral with respect to its effect on mortality, except in decreasing the risk of mortality in the cohort born from 1926–1935. Overall, these results suggest that separating the effects of adoption on mortality by gender produces results that are consistent with the combined analysis.

## Discussion

Evolutionary perspectives have often focused on genetic relatedness as a predictor of adoptive placements [6,19–21] and care [3,23–25], typically finding that adoptive children fare worse than their biological counterparts. In testing this hypothesis, we failed to replicate similar findings showing higher mortality among adoptive children in historical Taiwan. Using a larger longitudinal dataset and controlling for covariates known or hypothesized to affect adoption status or mortality, we have shown that daughters adopted by Taiwanese during the Japanese colonial administrative period, who were not related to their adoptive parents, did not experience an increase in mortality relative to their biological counterparts. In association with gender (female; Table 2 and S4 Table), increasing prevalence of minor marriage (S4 and S5 Tables), and calendar time (Table 2 and S4–S6 Tables), they may even have experienced an advantage in survivorship, as suggested by the unadjusted data (Figs 1 and 2 and S2 and S6 Tables). To our knowledge, these are the first analyses showing a protective effect of adoption on mortality in a non-Western setting, let alone a protective effect where adoption has often been viewed negatively. Using high-quality data and Cox proportional hazards analysis of adoption as a time-dependent covariate, we have attenuated as much as possible any selection bias that might otherwise have driven the mortality differences shown here between adopted daughters and other categories of children. Our results do not illuminate the specific pathways by which adoption lowered the risk of mortality in historical Taiwan, but we suggest that they are likely due to the specific kinship norms in this population that allowed adoptive parents to benefit from marriage of ADIL to co-resident sons and, possibly through this custom, to benefit from decreased frailty of ADIL relative to other children.

The first hypothesis suggested by our analyses is that lower mortality reflects parental investment in adopted children and ADIL in particular. Ethnographic and demographic evidence are consistent with the maltreatment of ADIL in their adoptive households (e.g., [14,15,41]), but have also noted that ADIL acquired at young ages were breastfed by their adoptive mothers and subsequently developed life-long (if sometimes fraught) attachments to them [14,15,17]. More generally, adoptive parents reportedly derived several benefits from their ADIL, including labor, but perhaps more importantly, the latter’s contribution to lineage perpetuation without the economic costs or inter-generational strains associated with major marriage [14,15]. Indeed, from the perspective of kin selection theory, ADIL might be expected to receive better treatment than adoptive daughters, since the former resulted in biological, rather than social, perpetuation of the lineage. ADIL may also have imposed fitness costs, however, if fertility associated with minor marriage was lower than fertility of major marriages [15]. Future analyses will attempt to confirm the result that minor marriage, through incest avoidance mechanisms (the “Westermarck effect”), is associated with decreased fertility, controlling for important covariates as we do here. If this result is upheld, it is still plausible that parents with ADIL have greater numbers of grandchildren than parents who do not (e.g., because ADIL contribute to the fertility of their co-resident in-laws). To our knowledge, this has not yet been explored in any population in which Westermarck effects have been proposed to explain decreased fertility among married couples. Regardless, a mortality advantage associated with ADIL need not contradict kin selection theory if the benefits associated with adoption outweighed its potential costs. Future analyses could explore this issue by comparing the reproductive success of mothers who adopt ADIL to those who do not. At the very least, our results suggest that adopted children did not suffer a mortality disadvantage compared to biological children reared in their natal households. The voluntary nature of most adoptions in Taiwan during this time period as described in the introduction (i.e., as opposed to adopting a child due to the death of a sibling) suggests that parents were ready to invest in adopted children in order for those children to fulfill their normative purposes. It is possible that ethnographic descriptions of maltreatment of adopted children accurately reflected community norms surrounding adoption, but that parenting behavior, on average, was inconsistent with those norms.

It is also conceivable that adopted children were less frail than biological ones. One study of parental investment and health outcomes among children in the contemporary People’s Republic of China (PRC) has shown that even though parental investment measures (education, immunization) were lower for adopted children, their health outcomes were nonetheless similar to those of biological children [56]. Adopted children’s circumstances are often, but not always, improved via adoption, and they often catch up to biological peers where initial differences are apparent, indicating resiliency among adopted children [2]. Where intermediaries were involved in selecting adoptive children for adoptive parents, such children would often have been vetted to determine appropriateness of the match, if not also for health and vitality [41]. Though we have limited ethnographic evidence to support increased robustness of ADIL, Brown has noted improved survivorship of AD in her ethnographic Taiwan sample relative to the general population and surprisingly little vetting by intermediaries [50]. The effects of adoption were mostly upheld, if attenuated, in a dataset excluding adult individuals (S5 Table), suggesting that parental investment and decreased frailty of adopted children (especially ADIL) may both have contributed to their decreased mortality. The frailty hypothesis suggested by our analyses can only be adequately tested using data where health at time of adoption is known.

The pattern of mortality that we have described appears normal for non-adopted males in this population [41,46], but the difference in infant mortality of adopted girls compared to other categories of children is striking and unexpected based on previous reports. This result is unlikely to be due to systematic under-reporting of deaths of non-adopted girls (e.g., due to infanticide), given the Taiwanese registers’ extraordinary accuracy [14,41–43,45,46]. Even if female deaths *were* under-reported (contra [14,41–43,45,46]), it could not explain the persistent mortality advantage at older ages apparent among adopted daughters (Fig 1). Thus, we eliminate female infanticide or neglect as potential sources of unperceived bias. If our hypothesis is borne out that parental investment in ADIL was similar or superior to investment in biological children, it would contradict stated practices and previous evidence of Taiwanese during the examined time-period. This would not be the first case where actual practices differed from stated practices, including within this population [14,17,41]. The discrepancies between previous results and ours are likely due to differences in statistical methods and the commonly noted divergence between measurable outcomes and stated practices.

The relationship depicted here between adoption and mortality does not explicate the precise pathways by which adoption acts to lower mortality. In particular, the majority of our control variables depict circumstances of the indexed individuals in their natal households (at the time of their birth). While this provides an apt comparison of the fates of those who remained in their natal households versus those who were adopted out, it does not explain what features of adoption were likely to have been protective. Adoption could act on its own as a proxy for parental investment; alternatively, it may be associated with other characteristics in the adoptive household (e.g., lower birth order, smaller household size) that direct resources to adopted children in line with more general norms of, or available resources for, investing in children. While this issue is common to all longitudinal studies using fixed (i.e., not time-dependent) covariates, future analyses exploring the circumstances surrounding adoption will provide more resolution on the precise pathways leading to the association shown here.

Both biological and adoptive parents have been shown to be highly strategic in decisions of how to solicit or provide substitute care [6,7]. If Taiwanese did indeed invest more in ADIL or selected ADIL for lack of frailty, then our results provide further evidence suggestive of strategic decision making among adoptive parents by showing that ADIL, who provided labor and an inexpensive means of lineage perpetuation, suffered similar or lower mortality compared to adopted sons and biological children remaining in their natal households. This result also underscores that while kin selection may explain many patterns affecting adoptive practices, the associated costs and benefits, mediated by socio-structural context, are of at least equal importance [6,8]. Furthermore, the different constraints and motivations generated by such contexts are likely to be associated with differences in parental investment and its outcomes. Adoption is a human universal that is likely to be adaptive under many circumstances [8], particularly for adoptive children whose position may be improved as a result of adoption relative to their natal circumstances or the circumstances around the time of their adoption [8,21]. Genetic relatedness is one of many predictors of adoptive behavior across species [33], suggesting deep evolutionary roots to adoptive plasticity. Such plasticity underscores the importance of socio-ecological context in determining the predictors of adoption and its associated outcomes [57–60].

## Conclusion

The unusual system of minor marriage has long been considered to be of little economic or demographic benefit to adoptive parents, and its association with maltreatment and poor outcomes of adoptive children has been used to bolster kin selection arguments that link the degree of genetic relatedness to parental investment. However, we have shown that adoption, and likely minor marriage adoption in particular, was protective or at least neutral with respect to its effects on mortality, and that this effect is consistent with either decreased frailty of adopted children compared to those who remained in their natal households or with increased parental investment by adoptive parents. If parental investment in adoptive children contributed to its effects on decreasing mortality, then something other than genetic relatedness must explain why. We have proposed that the socio-structural context in historical Taiwan may have favored more intensive parental investment than has previously been described; this should be confirmed using other data, both qualitative and quantitative.

Minor marriage, once nearly ubiquitous in Taiwan, no longer occurs. Despite ethnographic and now quantitative evidence of some advantages of this system, it was eliminated as a means of forming new marriages after 1950. The particulars of this unusual system may make it a poor candidate for generalizing across systems of adoption; however, it does reinforce the context-specific nature of adoption practices and their outcomes. Thus, current policy in the United States and elsewhere, dictating a preference for placement of maltreated children among kin [5] may also benefit from considering the specific circumstances that favor adoption (e.g., death of a family member, infertility, etc.) in any given case. Moreover, where such factors have emerged as equally or more important to adoptive and fostering outcomes (e.g., the particular histories of children involved [24] and socio-demographic characteristics such as sex, race, and socio-economic status of biological and adoptive parents [2]), such findings have occasionally led to the rejection of biological notions of adoption (e.g., [58]). They are, however, compatible with a broader evolutionary framework, which takes into account how socio-structural factors alter costs and benefits of family care [6,7,61,62]. Our work suggests that evolutionary arguments linking genealogical relatedness to the well-being of fostered and adopted children should also incorporate underlying socio-structural variables (e.g., cultural practices, socio-economic status, education, etc.—namely the social and cultural niche) [61,62] that mediate the association between degree of genetic relatedness and adoptive outcomes. The result will be a fuller understanding of how more general evolutionary arguments can explain adoption and inform related policies [5,63,64].

## Supporting Information

S1 Fig Map of Taiwan, showing site locations. (PNG)

S2 Fig Survival plots, by gender, adoption status, and birth cohort. The plots show that the effect of adoption on survivorship was increasingly protective over time. The difference is most notable in the cohort born from 1925–1936, but the survivorship of adopted individuals seemed to improve more than survivorship of non-adopted children as time went on.
(PDF)

S1 Table Taiwanese site description and sample size. (DOCX)

S2 Table Observed age-specific mortality rates (ASMR) by standard age intervals. (DOCX)

S3 Table Abridged life tables. (DOCX)

S4 Table The effects of covariates on the instantaneous hazard of mortality (all ages). (DOCX)

S5 Table The effects of covariates on the instantaneous hazard of mortality through age 15. (DOCX)

S6 Table The effects of covariates on the instantaneous hazard of mortality at all ages, by gender. (DOCX)
