# Supplementary figures and images for: Adoption Does Not Increase the Risk of Mortality among Taiwanese Girls in a Longitudinal Analysis
Source: PLoS One. 2015 Apr 29;10(4):e0122867. doi: 10.1371/journal.pone.0122867 (PMC4414473; doi:10.1371/journal.pone.0122867)

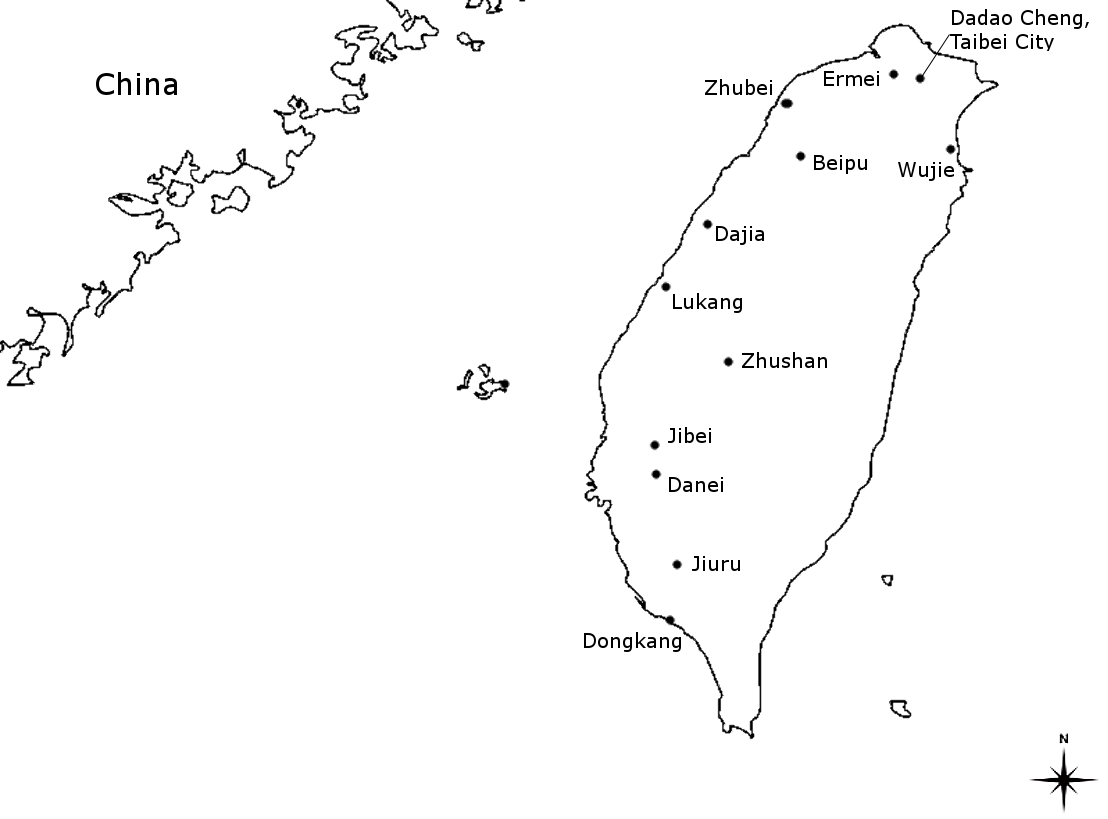

Supplement: S1 Fig — (PNG) [file pone.0122867.s001.png]

**Born 1906-1915**

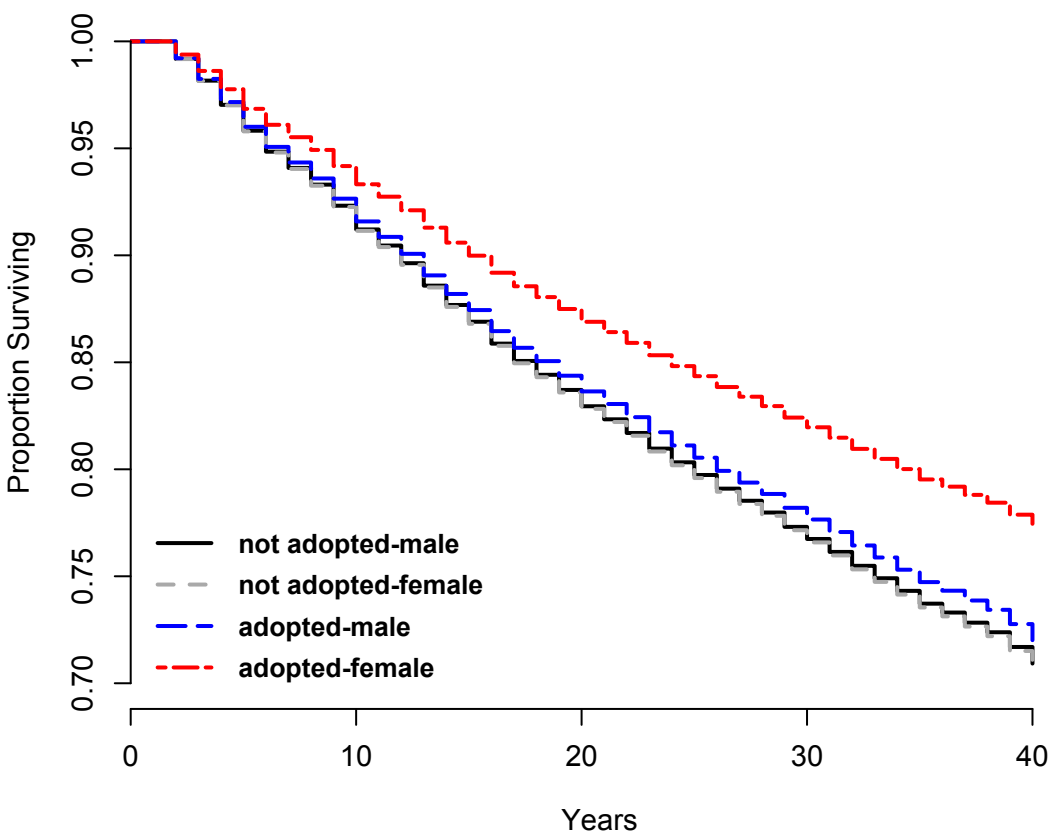

**Born 1916-1925**

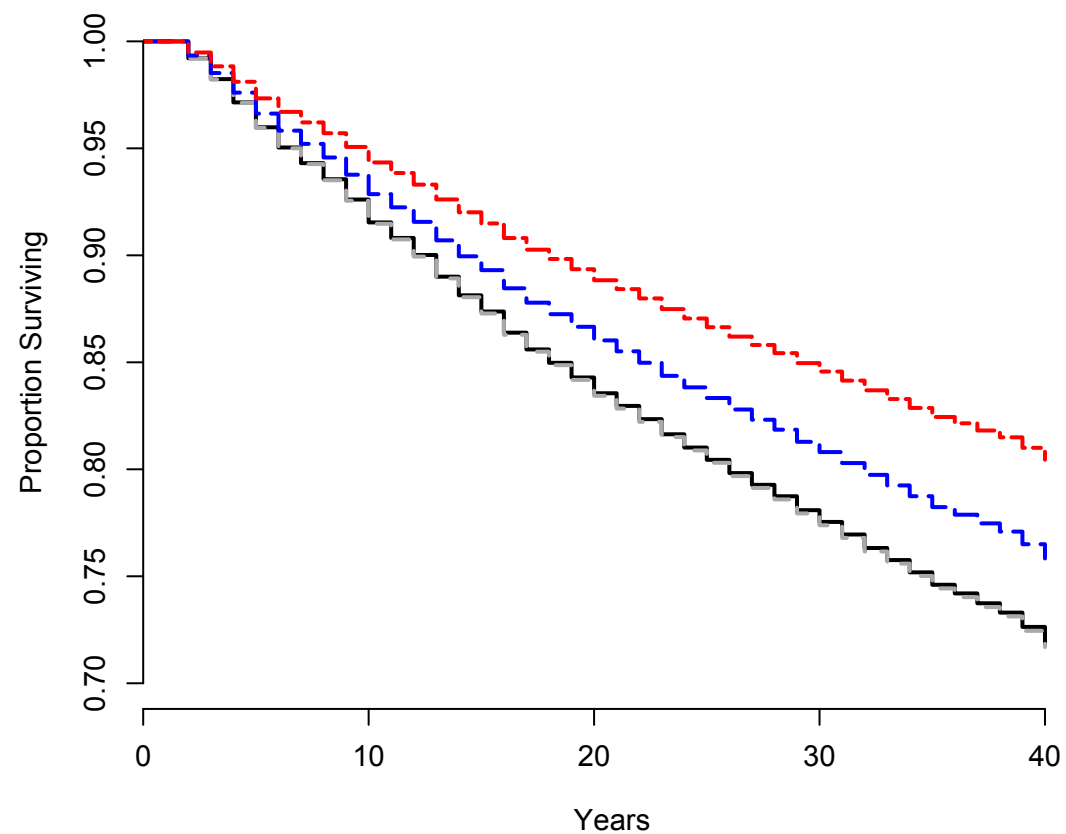

**Born 1926-1935**

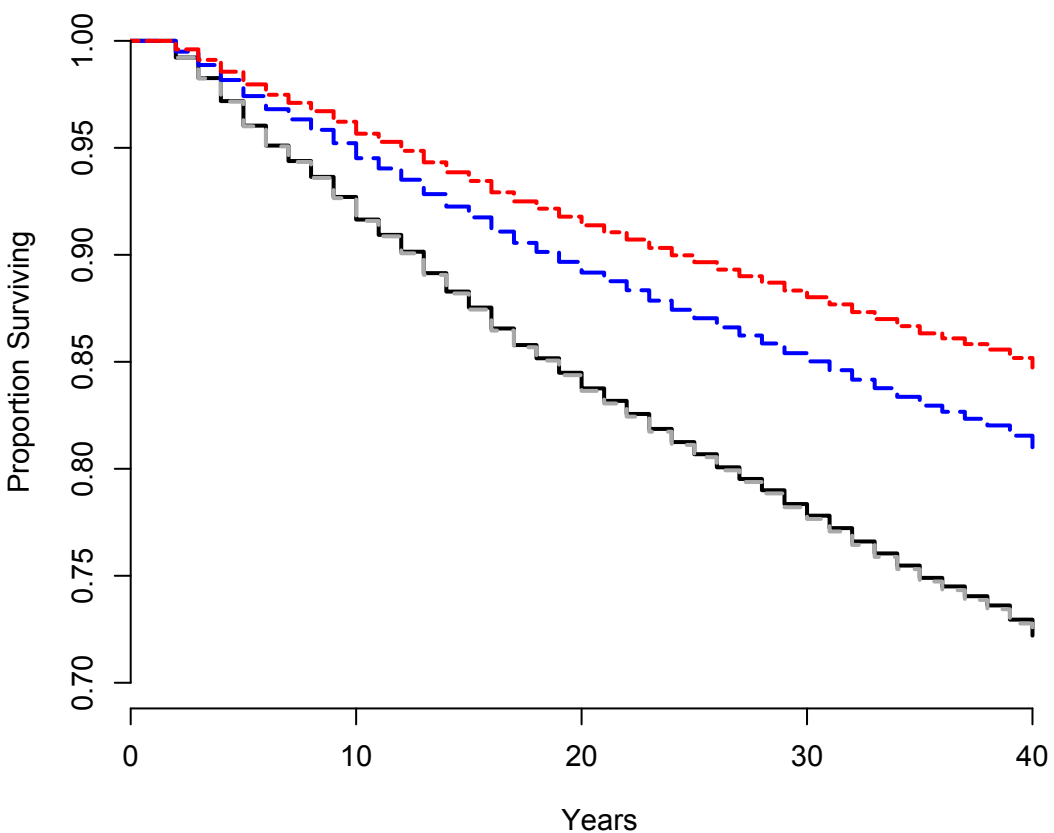

**Born 1936-1945**

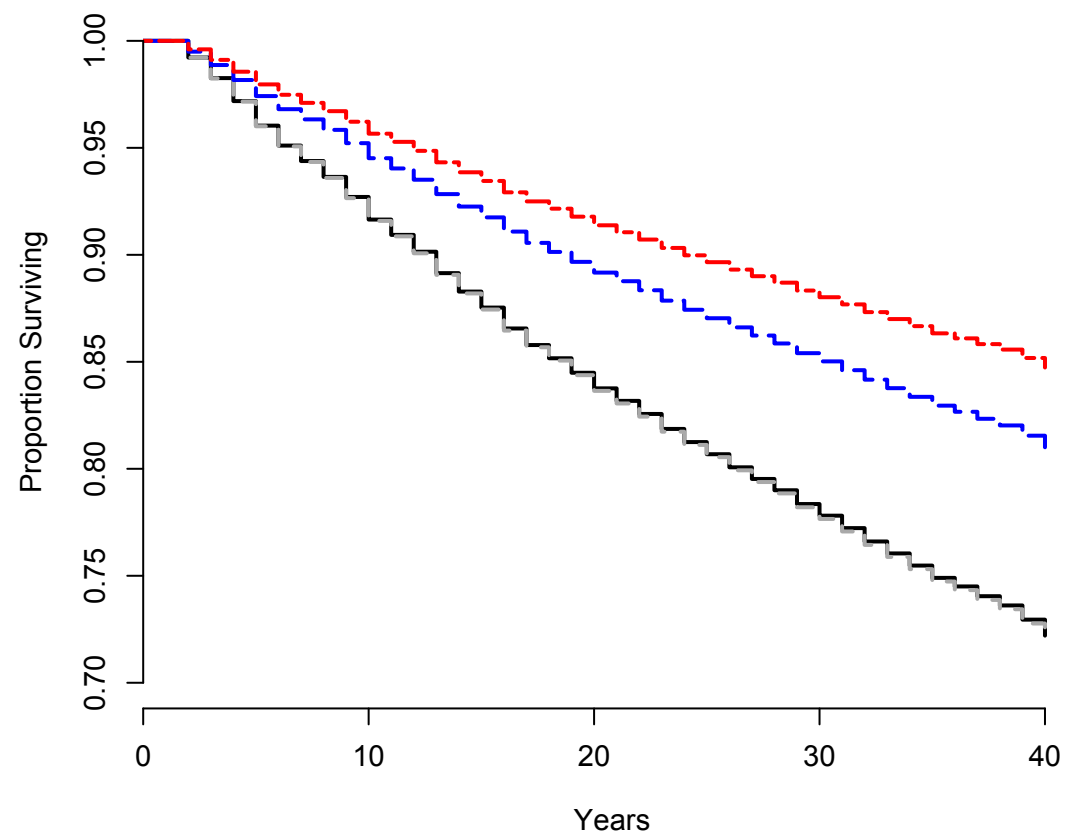

Supplement: S2 Fig — The plots show that the effect of adoption on survivorship was increasingly protective over time. The difference is most notable in the cohort born from 1925–1936, but the survivorship of adopted individuals seemed to improve more than survivorship of non-adopted children as time went on. (PDF) [file pone.0122867.s002.pdf]
