# Supplementary material for: Adoption Does Not Increase the Risk of Mortality among Taiwanese Girls in a Longitudinal Analysis
Source: PLoS One. 2015 Apr 29;10(4):e0122867. doi: 10.1371/journal.pone.0122867 (PMC4414473; doi:10.1371/journal.pone.0122867)
Supplement: S1 Table — (DOCX) [file pone.0122867.s003.docx]

| **Table S1. Taiwanese site description and sample size^a^** | | | | |
| --- | --- | --- | --- | --- |
| City | Region | Urban/Rural | Level of ADIL Adoption | Sample (N Inds) |
| Dadao Cheng neighborhood, Taibei City  (大稻埕/台北) | north | urban | moderate | 6,819 |
| Beipu, Xinzhu County  (北埔/新竹) | north | rural | high | 1,963 |
| Zhubei, Xinzhu County  (竹北/新竹) | north | rural | high | 10,258 |
| Ermei, Xinzhu County  (峨眉/新竹) | north | rural | high | 7,492 |
| Wujie, Yilan County  (五結/宜蘭) | north(east) | rural | high | 8,060 |
| Dajia, Taizhong County  (大甲/台中) | central | town | moderate | 1,814 |
| Lukang, Zhanghua County (鹿港/彰化) | central | town | moderate | 5,616 |
| Zhushan, Nantou County  (竹山/南投) | central | rural | moderate | 7,594 |
| Jibei, Tainan County  (吉貝耍/台南) | south | rural | low | 1,994 |
| Jiuru, Pingdong County  (九如/屏東) | south | rural | low | 2,527 |
| Danei, Tainan County  (大內/台南) | south | rural | low | 13,034 |
| Dongkang, Pingdong County (東港/屏東) | south | town | low | 3,171 |
| Huxi, Penghu County  (湖西/澎湖) | island | rural | highest | 4,350 |
| ^a^ “Levels” of minor marriage (ADIL) adoption based on [40]: high is ≥ 40%; moderate is 30-40%; low is 0-20%; highest is 71.1% of daughters adopted out by age 15. | | | | |
