# Supplementary material for: Adoption Does Not Increase the Risk of Mortality among Taiwanese Girls in a Longitudinal Analysis
Source: PLoS One. 2015 Apr 29;10(4):e0122867. doi: 10.1371/journal.pone.0122867 (PMC4414473; doi:10.1371/journal.pone.0122867)
Supplement: S2 Table — (DOCX) [file pone.0122867.s004.docx]

| **Table S2. Observed age-specific mortality rate (ASMR)^a^ by standard age intervals** | | | | | |
| --- | --- | --- | --- | --- | --- |
| age interval^b^ | total | male | female | not adopted | adopted |
| 0-1 | 0.173 | 0.187 | 0.159 | 0.193 | 0.013 |
| 1-4 | 0.033 | 0.032 | 0.035 | 0.350 | 0.017 |
| 5-9 | 0.006 | 0.006 | 0.007 | 0.006 | 0.004 |
| 10-14 | 0.003 | 0.003 | 0.003 | 0.003 | 0.002 |
| 15-19 | 0.004 | 0.005 | 0.003 | 0.004 | 0.002 |
| 20-24 | 0.009 | 0.010 | 0.006 | 0.009 | 0.005 |
| 25-29 | 0.009 | 0.010 | 0.009 | 0.009 | 0.008 |
| 30-34 | 0.011 | 0.012 | 0.009 | 0.011 | 0.011 |
| 35-39 | 0.018 | 0.020 | 0.014 | 0.020 | 0.015 |

| age interval |  | male,  not adopted | male,  adopted | female,  not adopted | female,  adopted |
| --- | --- | --- | --- | --- | --- |
| 0-1 |  | 0.193 | 0.018 | 0.192 | 0.012 |
| 1-4 |  | 0.032 | 0.019 | 0.039 | 0.016 |
| 5-9 |  | 0.006 | 0.002 | 0.007 | 0.004 |
| 10-14 |  | 0.003 | 0.001 | 0.003 | 0.002 |
| 15-19 |  | 0.005 | 0.002 | 0.004 | 0.002 |
| 20-24 |  | 0.010 | 0.011 | 0.007 | 0.003 |
| 25-29 |  | 0.010 | 0.010 | 0.010 | 0.008 |
| 30-34 |  | 0.012 | 0.020 | 0.010 | 0.007 |
| 35-39 |  | 0.020 | 0.016 | 0.014 | 0.015 |

^a^ Calculated as number of death events in an age interval/person-years of exposure to risk

^b^ Years
