# Supplementary material for: Adoption Does Not Increase the Risk of Mortality among Taiwanese Girls in a Longitudinal Analysis
Source: PLoS One. 2015 Apr 29;10(4):e0122867. doi: 10.1371/journal.pone.0122867 (PMC4414473; doi:10.1371/journal.pone.0122867)
Supplement: S3 Table — (DOCX) [file pone.0122867.s005.docx]

| **Table S3. Abridged life tables^a^** | | | | | | | | |
| --- | --- | --- | --- | --- | --- | --- | --- | --- |
| *x* | *_n_a_x_* | *_n_m_x_* | *_n_q_x_* | *l_x_* | *_n_d_x_* | *_n_L_x_* | *T_x_* | *e_x_* |
| Male | | | | | | | | |
| 0 | 0.388 | 0.187 | 0.168 | 1.000 | 0.168 | 0.935 | 37.0 | 37.0 |
| 1 | 1.5 | 0.032 | 0.117 | 0.832 | 0.098 | 3.182 | 36.0 | 43.3 |
| 5 | 2.5 | 0.006 | 0.028 | 0.734 | 0.021 | 3.621 | 32.9 | 44.8 |
| 10 | 2.5 | 0.003 | 0.017 | 0.714 | 0.012 | 3.540 | 29.2 | 41.0 |
| 15 | 2.5 | 0.005 | 0.024 | 0.702 | 0.017 | 3.469 | 25.7 | 36.6 |
| 20 | 2.5 | 0.010 | 0.047 | 0.686 | 0.032 | 3.347 | 22.2 | 32.4 |
| 25 | 2.5 | 0.010 | 0.047 | 0.653 | 0.031 | 3.190 | 18.9 | 28.9 |
| 30 | 2.5 | 0.012 | 0.059 | 0.623 | 0.037 | 3.022 | 15.7 | 25.2 |
| 35 | 2.5 | 0.020 | 0.095 | 0.586 | 0.056 | 2.791 | 12.7 | 21.7 |
| 40 | 2.5 | 0.027 | 0.127 | 0.530 | 0.068 | 2.483 | 9.92 | 18.7 |
| 45 | 2.5 | 0.034 | 0.156 | 0.463 | 0.072 | 2.133 | 7.44 | 16.0 |
| 50 | 2.5 | 0.045 | 0.202 | 0.390 | 0.079 | 1.755 | 5.31 | 13.6 |
| 55 | 2.5 | 0.055 | 0.244 | 0.312 | 0.076 | 1.368 | 3.55 | 11.4 |
| 60 | 2.5 | 0.077 | 0.324 | 0.236 | 0.076 | 0.987 | 2.18 | 9.27 |
| 65 | 2.5 | 0.105 | 0.417 | 0.159 | 0.066 | 0.630 | 1.19 | 7.52 |
| 70 | 2.5 | 0.140 | 0.519 | 0.093 | 0.048 | 0.343 | 0.56 | 6.13 |
| 75 | 2.5 | 0.179 | 0.619 | 0.045 | 0.028 | 0.154 | 0.22 | 5.05 |
| 80 | 2.5 | 0.223 | 0.716 | 0.017 | 0.012 | 0.055 | 0.07 | 4.20 |
| 85 | 3.493 | 0.286 | 1 | 0.005 | 0.005 | 0.017 | 0.01 | 3.49 |
| Female | | | | | | | | |
| 0 | 0.339 | 0.159 | 0.143 | 1.000 | 0.143 | 0.951 | 40.1 | 40.1 |
| 1 | 1.5 | 0.035 | 0.130 | 0.857 | 0.111 | 3.259 | 39.1 | 45.7 |
| 5 | 2.5 | 0.007 | 0.032 | 0.745 | 0.024 | 3.666 | 35.9 | 48.2 |
| 10 | 2.5 | 0.003 | 0.013 | 0.721 | 0.009 | 3.583 | 32.2 | 44.7 |
| 15 | 2.5 | 0.003 | 0.017 | 0.712 | 0.012 | 3.529 | 28.6 | 40.2 |
| 20 | 2.5 | 0.006 | 0.030 | 0.700 | 0.021 | 3.446 | 25.1 | 35.9 |
| 25 | 2.5 | 0.009 | 0.046 | 0.679 | 0.031 | 3.316 | 21.7 | 31.9 |
| 30 | 2.5 | 0.009 | 0.045 | 0.648 | 0.029 | 3.166 | 18.3 | 28.4 |
| 35 | 2.5 | 0.014 | 0.070 | 0.619 | 0.043 | 2.986 | 15.2 | 24.6 |
| 40 | 2.5 | 0.023 | 0.109 | 0.576 | 0.062 | 2.722 | 12.2 | 21.2 |
| 45 | 2.5 | 0.027 | 0.126 | 0.513 | 0.065 | 2.404 | 9.52 | 18.5 |
| 50 | 2.5 | 0.034 | 0.157 | 0.449 | 0.070 | 2.066 | 7.11 | 15.8 |
| 55 | 2.5 | 0.045 | 0.202 | 0.378 | 0.076 | 1.699 | 5.05 | 13.3 |
| 60 | 2.5 | 0.059 | 0.259 | 0.302 | 0.078 | 1.313 | 3.35 | 11.1 |
| 65 | 2.5 | 0.078 | 0.328 | 0.224 | 0.073 | 0.935 | 2.03 | 9.11 |
| 70 | 2.5 | 0.106 | 0.419 | 0.150 | 0.063 | 0.594 | 1.10 | 7.33 |
| 75 | 2.5 | 0.146 | 0.535 | 0.087 | 0.047 | 0.320 | 0.50 | 5.81 |
| 80 | 2.5 | 0.194 | 0.654 | 0.041 | 0.027 | 0.137 | 0.18 | 4.63 |
| 85 | 3.659 | 0.273 | 1 | 0.014 | 0.014 | 0.051 | 0.05 | 3.65 |
| Not Adopted | | | | | | | | |
| 0 | 0.397 | 0.192 | 0.172 | 1 | 0.172 | 0.931 | 36.5 | 36.5 |
| 1 | 1.5 | 0.035 | 0.128 | 0.827 | 0.106 | 3.149 | 35.5 | 43.0 |
| 5 | 2.5 | 0.006 | 0.030 | 0.720 | 0.022 | 3.548 | 32.4 | 45.0 |
| 10 | 2.5 | 0.003 | 0.015 | 0.698 | 0.010 | 3.465 | 28.9 | 41.3 |
| 15 | 2.5 | 0.004 | 0.022 | 0.687 | 0.015 | 3.400 | 25.4 | 36.9 |
| 20 | 2.5 | 0.008 | 0.043 | 0.672 | 0.029 | 3.289 | 22.0 | 32.7 |
| 25 | 2.5 | 0.009 | 0.047 | 0.643 | 0.030 | 3.140 | 18.7 | 29.1 |
| 30 | 2.5 | 0.011 | 0.055 | 0.612 | 0.033 | 2.980 | 15.6 | 25.4 |
| 35 | 2.5 | 0.018 | 0.090 | 0.579 | 0.052 | 2.764 | 12.6 | 21.7 |
| 40 | 2.5 | 0.027 | 0.127 | 0.526 | 0.067 | 2.466 | 9.85 | 18.7 |
| 45 | 2.5 | 0.033 | 0.156 | 0.459 | 0.071 | 2.118 | 7.39 | 16.0 |
| 50 | 2.5 | 0.044 | 0.201 | 0.387 | 0.078 | 1.743 | 5.27 | 13.6 |
| 55 | 2.5 | 0.055 | 0.243 | 0.309 | 0.075 | 1.359 | 3.53 | 11.4 |
| 60 | 2.5 | 0.077 | 0.324 | 0.234 | 0.075 | 0.980 | 2.17 | 9.27 |
| 65 | 2.5 | 0.105 | 0.417 | 0.158 | 0.066 | 0.625 | 1.19 | 7.52 |
| 70 | 2.5 | 0.140 | 0.519 | 0.092 | 0.047 | 0.341 | 0.56 | 6.13 |
| 75 | 2.5 | 0.179 | 0.618 | 0.044 | 0.027 | 0.152 | 0.22 | 5.05 |
| 80 | 2.5 | 0.222 | 0.715 | 0.016 | 0.012 | 0.054 | 0.07 | 4.20 |
| 85 | 3.493 | 0.286 | 1 | 0.004 | 0.004 | 0.016 | 0.01 | 3.49 |
| Adopted | | | | | | | | |
| 0 | 0.09 | 0.013 | 0.013 | 1 | 0.013 | 0.998 | 52.5 | 52.5 |
| 1 | 1.5 | 0.016 | 0.063 | 0.986 | 0.062 | 3.852 | 51.5 | 52.2 |
| 5 | 2.5 | 0.004 | 0.019 | 0.923 | 0.018 | 4.573 | 47.6 | 51.6 |
| 10 | 2.5 | 0.001 | 0.009 | 0.905 | 0.008 | 4.505 | 43.1 | 47.6 |
| 15 | 2.5 | 0.002 | 0.010 | 0.896 | 0.009 | 4.460 | 38.6 | 43.0 |
| 20 | 2.5 | 0.005 | 0.025 | 0.887 | 0.022 | 4.378 | 34.1 | 38.4 |
| 25 | 2.5 | 0.008 | 0.042 | 0.864 | 0.036 | 4.229 | 29.7 | 34.4 |
| 30 | 2.5 | 0.011 | 0.054 | 0.827 | 0.045 | 4.024 | 25.5 | 30.8 |
| 35 | 2.5 | 0.015 | 0.073 | 0.782 | 0.057 | 3.767 | 21.5 | 27.4 |
| 40 | 2.5 | 0.015 | 0.073 | 0.724 | 0.053 | 3.489 | 17.7 | 24.4 |
| 45 | 2.5 | 0.019 | 0.090 | 0.671 | 0.060 | 3.203 | 14.2 | 21.2 |
| 50 | 2.5 | 0.025 | 0.118 | 0.610 | 0.072 | 2.870 | 11.0 | 18.1 |
| 55 | 2.5 | 0.034 | 0.158 | 0.538 | 0.085 | 2.477 | 8.17 | 15.1 |
| 60 | 2.5 | 0.046 | 0.209 | 0.452 | 0.094 | 2.027 | 5.69 | 12.5 |
| 65 | 2.5 | 0.063 | 0.275 | 0.358 | 0.098 | 1.543 | 3.67 | 10.2 |
| 70 | 2.5 | 0.088 | 0.363 | 0.259 | 0.094 | 1.060 | 2.12 | 8.20 |
| 75 | 2.5 | 0.124 | 0.475 | 0.164 | 0.078 | 0.628 | 1.06 | 6.47 |
| 80 | 2.5 | 0.172 | 0.602 | 0.086 | 0.052 | 0.302 | 0.43 | 5.06 |
| 85 | 3.95 | 0.252 | 1 | 0.034 | 0.034 | 0.136 | 0.13 | 3.95 |
| Male, Not Adopted | | | | | | | | |
| 0 | 0.40 | 0.193 | 0.173 | 1.000 | 0.173 | 0.931 | 36.752 | 36.75 |
| 1 | 1.5 | 0.032 | 0.119 | 0.827 | 0.098 | 3.162 | 35.821 | 43.30 |
| 5 | 2.5 | 0.006 | 0.028 | 0.729 | 0.021 | 3.594 | 32.659 | 44.79 |
| 10 | 2.5 | 0.003 | 0.017 | 0.708 | 0.012 | 3.512 | 29.065 | 41.03 |
| 15 | 2.5 | 0.005 | 0.024 | 0.697 | 0.017 | 3.441 | 25.553 | 36.69 |
| 20 | 2.5 | 0.010 | 0.047 | 0.680 | 0.032 | 3.320 | 22.112 | 32.53 |
| 25 | 2.5 | 0.010 | 0.047 | 0.648 | 0.030 | 3.164 | 18.793 | 29.00 |
| 30 | 2.5 | 0.012 | 0.057 | 0.618 | 0.035 | 3.001 | 15.628 | 25.30 |
| 35 | 2.5 | 0.020 | 0.096 | 0.582 | 0.056 | 2.773 | 12.628 | 21.68 |
| 40 | 2.5 | 0.027 | 0.127 | 0.527 | 0.067 | 2.465 | 9.855 | 18.72 |
| 45 | 2.5 | 0.034 | 0.156 | 0.460 | 0.072 | 2.118 | 7.390 | 16.08 |
| 50 | 2.5 | 0.045 | 0.202 | 0.388 | 0.078 | 1.743 | 5.272 | 13.60 |
| 55 | 2.5 | 0.055 | 0.244 | 0.309 | 0.075 | 1.359 | 3.530 | 11.41 |
| 60 | 2.5 | 0.077 | 0.324 | 0.234 | 0.076 | 0.980 | 2.171 | 9.28 |
| 65 | 2.5 | 0.105 | 0.417 | 0.158 | 0.066 | 0.626 | 1.191 | 7.53 |
| 70 | 2.5 | 0.140 | 0.519 | 0.092 | 0.048 | 0.341 | 0.565 | 6.13 |
| 75 | 2.5 | 0.179 | 0.619 | 0.044 | 0.027 | 0.153 | 0.224 | 5.05 |
| 80 | 2.5 | 0.223 | 0.716 | 0.017 | 0.012 | 0.054 | 0.071 | 4.20 |
| 85 | 3.49 | 0.286 | 1.000 | 0.005 | 0.005 | 0.017 | 0.017 | 3.49 |
| Male, Adopted | | | | | | | | |
| 0 | 0.10 | 0.018 | 0.018 | 1.000 | 0.018 | 0.998 | 48.818 | 48.82 |
| 1 | 1.5 | 0.019 | 0.071 | 0.982 | 0.070 | 3.823 | 47.820 | 48.70 |
| 5 | 2.5 | 0.002 | 0.012 | 0.912 | 0.011 | 4.533 | 43.997 | 48.23 |
| 10 | 2.5 | 0.001 | 0.007 | 0.901 | 0.007 | 4.489 | 39.464 | 43.79 |
| 15 | 2.5 | 0.002 | 0.012 | 0.895 | 0.011 | 4.445 | 34.975 | 39.10 |
| 20 | 2.5 | 0.011 | 0.052 | 0.884 | 0.046 | 4.303 | 30.529 | 34.55 |
| 25 | 2.5 | 0.010 | 0.051 | 0.838 | 0.043 | 4.081 | 26.227 | 31.31 |
| 30 | 2.5 | 0.020 | 0.097 | 0.795 | 0.077 | 3.782 | 22.145 | 27.86 |
| 35 | 2.5 | 0.016 | 0.077 | 0.718 | 0.055 | 3.452 | 18.364 | 25.58 |
| 40 | 2.5 | 0.016 | 0.078 | 0.663 | 0.051 | 3.186 | 14.912 | 22.50 |
| 45 | 2.5 | 0.022 | 0.102 | 0.611 | 0.063 | 2.900 | 11.726 | 19.18 |
| 50 | 2.5 | 0.031 | 0.143 | 0.549 | 0.078 | 2.548 | 8.826 | 16.08 |
| 55 | 2.5 | 0.041 | 0.184 | 0.470 | 0.087 | 2.135 | 6.278 | 13.35 |
| 60 | 2.5 | 0.060 | 0.260 | 0.384 | 0.100 | 1.669 | 4.143 | 10.80 |
| 65 | 2.5 | 0.084 | 0.348 | 0.284 | 0.099 | 1.173 | 2.475 | 8.72 |
| 70 | 2.5 | 0.115 | 0.448 | 0.185 | 0.083 | 0.718 | 1.302 | 7.03 |
| 75 | 2.5 | 0.152 | 0.551 | 0.102 | 0.056 | 0.370 | 0.584 | 5.71 |
| 80 | 2.5 | 0.196 | 0.658 | 0.046 | 0.030 | 0.154 | 0.214 | 4.65 |
| 85 | 3.80 | 0.263 | 1.000 | 0.016 | 0.016 | 0.060 | 0.060 | 3.80 |
| Females, Not Adopted | | | | | | | | |
| 0 | 0.40 | 0.192 | 0.172 | 1.000 | 0.172 | 0.932 | 39.700 | 39.70 |
| 1 | 1.5 | 0.039 | 0.142 | 0.828 | 0.118 | 3.134 | 38.768 | 46.84 |
| 5 | 2.5 | 0.007 | 0.035 | 0.710 | 0.025 | 3.488 | 35.634 | 50.19 |
| 10 | 2.5 | 0.003 | 0.013 | 0.685 | 0.009 | 3.404 | 32.146 | 46.90 |
| 15 | 2.5 | 0.004 | 0.019 | 0.676 | 0.013 | 3.349 | 28.742 | 42.51 |
| 20 | 2.5 | 0.007 | 0.034 | 0.663 | 0.023 | 3.260 | 25.393 | 38.28 |
| 25 | 2.5 | 0.010 | 0.048 | 0.641 | 0.031 | 3.125 | 22.133 | 34.55 |
| 30 | 2.5 | 0.010 | 0.048 | 0.610 | 0.030 | 2.974 | 19.008 | 31.18 |
| 35 | 2.5 | 0.014 | 0.068 | 0.580 | 0.040 | 2.802 | 16.033 | 27.64 |
| 40 | 2.5 | 0.015 | 0.074 | 0.541 | 0.040 | 2.603 | 13.232 | 24.48 |
| 45 | 2.5 | 0.019 | 0.091 | 0.501 | 0.045 | 2.389 | 10.629 | 21.23 |
| 50 | 2.5 | 0.025 | 0.118 | 0.455 | 0.054 | 2.141 | 8.240 | 18.10 |
| 55 | 2.5 | 0.034 | 0.158 | 0.401 | 0.063 | 1.848 | 6.098 | 15.20 |
| 60 | 2.5 | 0.047 | 0.210 | 0.338 | 0.071 | 1.512 | 4.251 | 12.58 |
| 65 | 2.5 | 0.064 | 0.276 | 0.267 | 0.074 | 1.151 | 2.738 | 10.25 |
| 70 | 2.5 | 0.089 | 0.364 | 0.193 | 0.070 | 0.791 | 1.587 | 8.21 |
| 75 | 2.5 | 0.125 | 0.475 | 0.123 | 0.058 | 0.469 | 0.796 | 6.47 |
| 80 | 2.5 | 0.172 | 0.602 | 0.065 | 0.039 | 0.226 | 0.327 | 5.07 |
| 85 | 3.95 | 0.253 | 1.000 | 0.026 | 0.026 | 0.102 | 0.102 | 3.95 |
| Females, Adopted | | | | | | | | |
| 0 | 0.09 | 0.012 | 0.012 | 1.000 | 0.012 | 0.999 | 53.471 | 53.47 |
| 1 | 1.5 | 0.016 | 0.062 | 0.988 | 0.062 | 3.859 | 52.472 | 53.12 |
| 5 | 2.5 | 0.004 | 0.021 | 0.926 | 0.020 | 4.581 | 48.614 | 52.49 |
| 10 | 2.5 | 0.002 | 0.010 | 0.906 | 0.009 | 4.509 | 44.033 | 48.59 |
| 15 | 2.5 | 0.002 | 0.011 | 0.897 | 0.010 | 4.463 | 39.523 | 44.04 |
| 20 | 2.5 | 0.003 | 0.017 | 0.888 | 0.015 | 4.402 | 35.060 | 39.49 |
| 25 | 2.5 | 0.008 | 0.039 | 0.873 | 0.034 | 4.278 | 30.658 | 35.13 |
| 30 | 2.5 | 0.007 | 0.036 | 0.838 | 0.030 | 4.118 | 26.381 | 31.46 |
| 35 | 2.5 | 0.015 | 0.072 | 0.809 | 0.058 | 3.897 | 22.263 | 27.53 |
| 40 | 2.5 | 0.015 | 0.074 | 0.750 | 0.055 | 3.613 | 18.366 | 24.48 |
| 45 | 2.5 | 0.019 | 0.091 | 0.695 | 0.063 | 3.316 | 14.753 | 21.23 |
| 50 | 2.5 | 0.025 | 0.118 | 0.632 | 0.075 | 2.972 | 11.437 | 18.10 |
| 55 | 2.5 | 0.034 | 0.158 | 0.557 | 0.088 | 2.565 | 8.465 | 15.20 |
| 60 | 2.5 | 0.047 | 0.210 | 0.469 | 0.098 | 2.099 | 5.900 | 12.58 |
| 65 | 2.5 | 0.064 | 0.276 | 0.371 | 0.102 | 1.598 | 3.801 | 10.25 |
| 70 | 2.5 | 0.089 | 0.364 | 0.268 | 0.098 | 1.098 | 2.203 | 8.21 |
| 75 | 2.5 | 0.125 | 0.475 | 0.171 | 0.081 | 0.651 | 1.105 | 6.47 |
| 80 | 2.5 | 0.172 | 0.602 | 0.090 | 0.054 | 0.313 | 0.454 | 5.07 |
| 85 | 3.95 | 0.253 | 1.000 | 0.036 | 0.036 | 0.141 | 0.141 | 3.95 |

^a^Column headings are as follows (see ^2^): *x*, exact age at beginning of age interval, [*x*, *x*+*n*); *_n_a_x_*, number of years lived by individuals who die in the interval, adjusted for intervals 0 and 1; *_n_m_x_*, observed or UN life table death rate in interval; *_n_q_x_*, probability of dying in age interval; *l_x_*, cumulative probability of survival to exact age *x*; *_n_d_x_*, number of deaths in the interval; *_n_L_x_* person-years lived in the interval; *T_x_*, person-years of life remaining at exact age *x*; *e_x_*, expectation of remaining life at exact age *x*.
