# Supplementary material for: Adoption Does Not Increase the Risk of Mortality among Taiwanese Girls in a Longitudinal Analysis
Source: PLoS One. 2015 Apr 29;10(4):e0122867. doi: 10.1371/journal.pone.0122867 (PMC4414473; doi:10.1371/journal.pone.0122867)
Supplement: S5 Table — (DOCX) [file pone.0122867.s007.docx]

| **Table S5. The effects of covariates on the instantaneous hazard of mortality (through age 15)^§,^*** | | | |
| --- | --- | --- | --- |
|  | Beta^a^ | SE^b^ | P |
| Adopted | 0.10 | 0.14 | 0.433 |
| Gender^c^ | -0.00 | 0.04 | 0.977 |
| Age | -0.23 | 0.01 | <0.001*** |
| Age^-2^ | 4.22 | 0.17 | <0.001*** |
| Living birth order | 0.01 | 0.00 | 0.001*** |
| Craftsman^d^ | -0.03 | 0.06 | 0.665 |
| Laborer^d^ | 0.06 | 0.03 | 0.028* |
| Landlord^d^ | -0.13 | 0.11 | 0.274 |
| Merchant^d^ | -0.07 | 0.03 | 0.023* |
| Uxorilocal^e^ | 0.028 | 0.03 | 0.367 |
| Illegitimate^f^ | 0.07 | 0.05 | 0.142 |
| Moderate minor marriage^g^ | -0.06 | 0.03 | 0.020* |
| High minor marriage^g^ | -0.27 | 0.03 | <0.001*** |
| Birth cohort = 2^h^ | -0.20 | 0.06 | 0.001** |
| Birth cohort = 3^h^ | -0.13 | 0.10 | 0.205 |
| Birth cohort = 4^h^ | -0.04 | 0.13 | 0.771 |
| Adopted x cohort 2 | -0.17 | 0.12 | 0.152 |
| Adopted x cohort 3 | -0.48 | 0.14 | <0.001*** |
| Adopted x cohort 4 | -0.24 | 0.22 | 0.273 |
| Gender(M) x living birth order | -0.01 | 0.00 | 0.098 |
| Gender(M) x adopted | 0.10 | 0.14 | 0.447 |
| Adopted x moderate minor marriage | -0.30 | 0.15 | 0.047* |
| Adopted x high minor marriage | -0.30 | 0.14 | 0.027* |
| **^§^**Number of death events =9,790; number of records = 486,691; includes all individuals from 0.5 years to age 15.  *p-value ≤0.05, **≤0.01, ***≤0.001. Adoption is modeled as a time-dependent covariate. See text for details. N is reduced compared to total sample due to missingness.  ^a^Beta is the estimated coefficient of the relationship between a given independent variable (e.g., gender) and the outcome of interest (here, the log hazard of mortality); i.e., a one-unit change in the independent variable is associated with a Beta increase in the log hazard of dying at any time.  ^b^SE is standard error of the estimated Beta.  ^c^Reference category is female.  ^d^Reference category for head of household’s occupation is agriculture.  ^e^Reference category is not uxorilocally married.  ^f^Reference category is legitimate.  ^g^Reference category is low prevalence of minor marriage; based on Supplementary Table 1; see Supplementary Methods for details.  ^h^Reference category is birth cohort 1; see Supplementary Table 1. | | | |
