# Supplementary material for: Adoption Does Not Increase the Risk of Mortality among Taiwanese Girls in a Longitudinal Analysis
Source: PLoS One. 2015 Apr 29;10(4):e0122867. doi: 10.1371/journal.pone.0122867 (PMC4414473; doi:10.1371/journal.pone.0122867)
Supplement: S6 Table — (DOCX) [file pone.0122867.s008.docx]

| **Table S6 The effects of covariates on the instantaneous hazard of mortality at all ages, by gender^§,^*** | | | | | | | |
| --- | --- | --- | --- | --- | --- | --- | --- |
|  | Males | | |  | Females | | |
|  | Beta^a^ | SE^b^ | P |  | Beta^a^ | SE^b^ | P |
| Adopted | 0.00 | 0.02 | 0.737 |  | -0.11 | 0.15 | 0.941 |
| Age | -0.00 | 0.00 | 0.172 |  | -0.68 | 0.00 | <0.001*** |
| Age^-2^ | 9.69 | 0.02 | <0.001*** |  | 6.95 | 0.02 | <0.001*** |
| Living birth order | 0.00 | 0.00 | 0.161 |  | 0.01 | 0.00 | 0.000*** |
| Craftsman^c^ | -0.04 | 0.08 | 0.666 |  | -0.08 | 0.09 | 0.385 |
| Laborer^c^ | 0.09 | 0.03 | 0.012* |  | 0.03 | 0.04 | 0.494 |
| Landlord^c^ | -0.15 | 0.14 | 0.286 |  | -0.04 | 0.15 | 0.776 |
| Merchant^c^ | -0.03 | 0.04 | 0.498 |  | -0.11 | 0.04 | 0.009** |
| Uxorilocal^d^ | -0.00 | 0.04 | 0.865 |  | 0.09 | 0.04 | 0.034* |
| Illegitimate^e^ | 0.11 | 0.06 | 0.065 |  | 0.00 | 0.07 | 0.893 |
| Moderate minor marriage^f^ | -0.61 | 0.03 | 0.080 |  | -0.05 | 0.04 | 0.160 |
| High minor marriage^f^ | -0.32 | 0.03 | <0.001*** |  | -0.22 | 0.04 | <0.001*** |
| Birth cohort = 2^g^ | -0.05 | 0.07 | 0.444 |  | -0.05 | 0.08 | 0.563 |
| Birth cohort = 3^g^ | -0.09 | 0.11 | 0.409 |  | -0.04 | 0.13 | 0.767 |
| Birth cohort = 4^g^ | 0.06 | 0.16 | 0.724 |  | 0.25 | 0.18 | 0.164 |
| Adopted x cohort 2 | 0.23 | 0.22 | 0.316 |  | -0.27 | 0.13 | 0.035* |
| Adopted x cohort 3 | -0.76 | 0.35 | 0.029* |  | -0.40 | 0.14 | 0.005** |
| Adopted x cohort 4 | 0.16 | 0.41 | 0.706 |  | -0.34 | 0.25 | 0.176 |
| Adopted x moderate minor marriage | -0.27 | 0.26 | 0.282 |  | -0.09 | 0.17 | 0.571 |
| Adopted x high minor marriage | -0.18 | 0.24 | 0.442 |  | -0.24 | 0.15 | 0.115 |
| **^§^**Number of death events (male) =5,985; number of records = 369,440; Number of death events (female) = 4,978; number of records = 278,797; includes all individuals from 0.5 years.  *p-value ≤0.05, **≤0.01, ***≤0.001. Adoption is modeled as a time-dependent covariate. See text for details. N is reduced compared to total sample due to missingness.  ^a^Beta is the estimated coefficient of the relationship between a given independent variable (e.g., gender) and the outcome of interest (here, the log hazard of mortality); i.e., a one-unit change in the independent variable is associated with a Beta increase in the log hazard of dying at any time.  ^b^SE is standard error of the estimated Beta.  ^c^Reference category for head of household’s occupation is agriculture.  ^d^Reference category is not uxorilocally married.  ^e^Reference category is legitimate.  ^f^Reference category is low prevalence of minor marriage; based on Supplementary Table 1; see Supplementary Methods for details.  ^g^Reference category is birth cohort 1; see Supplementary Table 1. | | | | | | | |
